# Growth and Meat Quality of Grass Carp (*Ctenopharyngodon idellus*) Responded to Dietary Protein (Soybean Meal) Level Through the Muscle Metabolism and Gene Expression of Myosin Heavy Chains

**DOI:** 10.3389/fnut.2022.833924

**Published:** 2022-03-28

**Authors:** Xiaoyu Wang, Guoqing Liu, Shouqi Xie, Lei Pan, Qingsong Tan

**Affiliations:** ^1^College of Fisheries, Huazhong Agricultural University, Wuhan, China; ^2^Engineering Research Center of Green Development for Conventional Aquatic Biological Industry in the Yangtze River Economic Belt, Ministry of Education, Wuhan, China; ^3^Key Laboratory of Freshwater Animal Breeding, Ministry of Agriculture and Rural Affairs, Wuhan, China; ^4^Hubei Provincial Engineering Laboratory for Pond Aquaculture, Wuhan, China; ^5^State Key Laboratory of Freshwater Ecology and Biotechnology, Institute of Hydrobiology, Chinese Academy of Sciences, Wuhan, China; ^6^Faculty of Resources and Environmental Science, Hubei University, Wuhan, China

**Keywords:** soybean meal, grass carp, myosin heavy chain, meat quality, antioxidant capacity

## Abstract

The aim of this study was to investigate the effect of dietary protein level (soybean meal) on growth performance, flesh quality of grass carp, and the related molecular mechanisms. The results showed that appropriate dietary protein levels improved the growth performance, hardness, and pH of muscle while decreasing muscle crude lipid content and cooking loss and altering the antioxidant capacity and metabolic enzymes activities. In addition, appropriate dietary protein promoted the gene expression of *myhc-1, myhc*-*4, myf5, myod, myog*, and *fgf6a*, whereas inhibited that of *myhc-7, myhc*-*2, mrf4*, and *mstn*. Transcriptome profiling of muscle revealed that the flesh quality-specific differences were related to tight junctions and intramuscular fat (IMF) accumulation. GSEA showed that fatty acid metabolism and oxidative phosphorylation were downregulated in SM5 compared with SM1. To conclude, appropriate protein levels improved the growth and flesh quality by regulating muscle antioxidant capacity and gene expression of *myhc*s and fat metabolism-related signaling molecules.

## Introduction

Protein, the significant component in aquafeeds for aquatic animals' growth and energy source, is getting more attention to control its dietary inclusion due to the shortage of protein sources. Grass carp (*Ctenopharyngodon idellus*) is one of the most prevalent freshwater species in the intensive aquaculture system in China (1). The protein requirements of this fish were previously determined using semipure diets containing fishmeal or casein as a protein source (2, 3). However, optimal dietary protein levels varied according to the protein sources due to the difference in protein quality (4). Soybean meal (SM), which contains various antinutritional factors, is currently the primary plant protein source in commercial aquafeeds for herbivorous fish, and studies on the use of SM in a fish diet focused on the potential of SM to replace fishmeal or its impact on the health and immunity of fish (5). However, researchers paid less attention to the independent use of SM as a dietary protein source, which imposes the necessity of reassessment of the optimal dietary protein level when SM was used as the major protein source.

It has been suggested that the dietary use of alternative protein sources might bring a degradation of meat quality associated with changes in texture (4). Skeletal muscle, a heterogeneous tissue, is composed of fibers varying in metabolic, biochemical, and biophysical characteristics, which are closely related to the types of myosin heavy chain (*myhc*) (6). Not surprisingly, myosin heavy chains can have a profound influence on flesh quality (7). At present, four *myhc* isoforms (*myhc-7, -4, -2*, and *-1*) have been identified in adult mammal skeletal muscle (6). Similarly, the polymorphisms of *myhc* isoform have been proven in fish skeletal muscles such as zebrafish (*Danio rerio*) (8), torafugu (*Takifugu rubripes*) (9), grass carp (10), and so on. It has also been evidenced that the fish muscle fibers containing unique *myhc* exhibit unusual characteristics such as fiber diameter, oxidative potential, and metabolic pattern (9). However, it is unclear to what extent dietary protein can improve the meat quality of grass carp, especially whether specific *myhc* is more likely to give better flesh quality characteristics?

Recently, we reported that dietary cottonseed meal replacement by distiller's dried grains with solubles (DDGS) induced changes in the texture, gene expression of *myhc* isoforms, along with muscle regulatory factors (MRFs) in grass carp muscle (10). The MRF family (*myod, myf5, myog*, and *mrf4*) as essential classic transcription activators is involved in the process of satellite cell activation during myogenesis and muscle regeneration (11). *Myod* and *myf5* are first expressed in myoblasts before differentiation (12), whereas *mrf4* and *myog* may be highly associated with myoblast differentiation and fusion in the later period forming muscle fibers (11). The transcript of *myod* may also be associated with the profile of *myhc* isoforms in fish (8).

Transcriptomic analysis based on RNA sequencing (RNA-seq) enables the accurate measurement of the changes in global gene expression profiles and comprehensively provides systematic networks about gene function, cell responses, and evolution, which has been widely applied to parse muscle development and integrally identify the regulatory genes associated with meat quality of grass carp (13). In addition, gene set enrichment analysis (GSEA) could highlight genes weakly connected to the phenotype through pathway analysis and has been widely used to exhibit significant differences in gene expression between samples (14).

Due to the important function of dietary protein in fish growth and meat quality, we hypothesized that optimal dietary protein level should be reassessed when soybean meal was used as a single protein source and that dietary protein level would regulate myocyte proliferation, hypertrophy, and myosin heavy chain isoform gene expression through MRFs and ultimately affect flesh quality. In this study, a growth trial by feeding juvenile grass carp with diets containing six levels of protein using soybean meal as a protein source was conducted, followed by a series of comprehensive physiological, biochemical, and transcriptome analyses to determine the optimal dietary soybean meal protein level and the related mechanism of dietary protein level on muscle texture.

## Materials and Methods

### Experimental Diets

The ingredients and approximate composition of the diets are presented in Appendix Table 1. Six experimental diets, namely, SM1, SM2, SM3, SM4, SM5, and SM6, respectively, were compounded using soybean meal as a protein source to obtain six protein levels (21, 25, 29, 33, 37, and 41%, respectively). The analyzed protein levels in the diets were 20.95, 24.83, 27.68, 32.26, 35.98, and 39.76%, respectively. Also, 0.1% yttrium oxide (Y_2_O_3_) as an indigestible marker for apparent digestibility coefficients (ADCs) determination was added to the experimental diets (15). Dietary ingredients were ground through an 80-mesh screen, weighed, and thoroughly mixed to homogeneity using a mixer (M-256, South China University of Technology, Guangzhou, China). The 2-mm-diameter pellets were produced using a thermal feed extruder (the moisture of meals: 21%, the extruder barrel temperature: 130°C), and then dried in an electric oven at 60°C for 30 min, and stored at −20°C until use.

### Fish and Experimental Management

Juvenile grass carp was obtained from Xinzhou Fisheries Co. Ltd. (Wuhan, China) and fed with a mixture of the six experimental diets with an equal percentage for 14 d before the feeding trial to adapt to the experimental conditions. After adaptation, fish (initial weight 6.80 ± 0.10 g) were randomly distributed into 18 tanks (300 L water) at a density of 30 fish per tank. Each dietary treatment was then randomly assigned to 3 tanks (replicates). During the 56-d feeding trial, fish were handfed to apparent satiation two times daily (8:30 am and 3:00 pm) with the experimental diets. After the fish stopped eating, the remained feed was collected by siphoning and dried at 60°C for 24 h to correct the feed intake. The water temperature was maintained at 29°C ± 1°C, pH was at 7.0 ± 0.3, and the dissolved oxygen was kept above 6.0 mg/L through constant aeration, and the photoperiod was 12 L: 12 D (15). All experiments were conducted following the guidelines for animal experimentation established by the Institutional Animal Care and Use Committee (IACUC) of Huazhong Agricultural University (Wuhan, China).

### Sample Collection

At the end of the feeding trial, fish fasted for 24 h. All fish from each tank were anesthetized with 100 mg L^−1^ tricaine methanesulfonate (MS-222, Sigma, St. Louis, Missouri, USA), counted, and collectively weighed. Six fish from each tank were randomly picked and divided into two parts, then pooled as a sample, and frozen at −20°C for proximate analysis of the whole body and muscle. Next, the dorsal white muscle of experimental fish in each tank was dissected and divided into three parts: one was fast frozen in liquid nitrogen and stored at −80°C for the analyses of metabolic and antioxidant enzyme activities, one was immediately immersed in liquid nitrogen and subsequently stored at −80°C prior to RNA extraction for muscle fiber-related gene expression analysis, and the other part was for the analysis of muscle physical and texture parameters. In addition, the dorsal muscle from SM1 and SM5 groups were prepared for transcriptomic analysis; that is, three random individuals in each tank were pooled as a sample and immediately immersed into liquid nitrogen for RNA extraction. White muscle specimens were also sampled and fixed in 4% buffered formalin for histomorphological observation.

### Analytical Determinations

#### Proximate Composition Assays

The chemical composition of diets, whole body, and muscle were assessed using the standard AOAC procedures (16). Briefly, moisture was determined by drying in a drying oven (DHG-9140A; Jinghong Laboratory Instrument Co., Ltd, Shanghai, China) at 105°C for 4 h until constant weight. Crude protein content (nitrogen × 6.25) was determined by the Kjeldahl method after sulfuric acid digestion using an automated Nitrogen Analyzer (SH220, Hannon Instruments Co. Ltd., Jinan, China). Crude lipid was extracted with ether by the method of Soxhlet, and the ash content was detected at 550°C for 12 h in a muffle furnace (SX2-4-10, Longkou City Electric Furnace Process Factory, Longkou, China).

#### Digestibility Measurements

The inductively coupled plasma atomic emission spectrophotometer (ICP; model: IRIS Advantage HR; Thermo Jarrell Ash Corporation, Boston, MA, USA) was used to detect the content of yttrium in both diets and feces after nitric acid digestion.

#### Determination of Muscle and Hepatopancreas Antioxidant Capacity

After thawing the samples on crushed ice, about 100 mg of white muscle and hepatopancreas samples was weighed from 3 fishes, respectively, mixed with normal saline at a volume ratio of 1:9, homogenized in the ice bath, and centrifuged at 2,500 × g (4°C, 10 min) to separate the supernatant. The supernatant from tissues was used for the determination of superoxide dismutase (SOD) activity (WST-1 method, Product NO., A001-3-2), total antioxidant capacity (T-AOC) activity (ABTS method, Product NO., A015-2-1), and malondialdehyde (MDA, TBA method, Product NO., A003-1-2) level using commercial kits (Nanjing Jiancheng Bioengineering Institute, Nanjing, China) according to the producer's manual.

#### Determination of Muscle Metabolic Enzymes

The activities of hexokinase (HK) (Product NO., A077-3) and lactate dehydrogenase (LDH) (Product NO., A020-2) in the dorsal white muscle were determined with commercial kits (Nanjing Jiancheng Bioengineering Institute, Nanjing, China) according to the manufacturer's manual.

#### Dorsal White Muscle Microstructure Analysis

White muscle fixed in 4% paraformaldehyde solution was dehydrated by several grades of ethyl alcohol and finally embedded in paraffin. Thick sections (7 μm) were prepared and then stained with hematoxylin and eosin (HE). The samples were observed by a light microscope equipped with the M Shot Image Analysis System (Micro-shot, Guangzhou, China). The ImagePro Plus (version 6.0) was used to measure the fiber diameters and calculate the average value.

#### Cooking Loss and Texture Profile Analysis

Fresh muscle pH was measured by a spear-type portable pH meter (HM-17MX, Toadkk, Japan). Cooking loss of fish muscle was measured as described by Abouel Azm et al. (15). After cooking loss measurement, the muscle was cut into a rectangular parallelepiped of 10 mm length, 10 mm width, and 5 mm height for texture measurement using a TA.XT Plus Texture Analyzer (Stable Micro Systems, Godalming, UK) equipped with a flat-bottomed cylindrical probe P/36R (20 mm diameter). Parameters for texture profile analysis (TPA) detection were set according to Abouel Azm et al. (15).

#### RNA Extraction and Illumina Sequencing

Total RNA was isolated from tissue samples using TRIzol Reagent (Invitrogen, CA, USA). The RNA concentration was measured using a NanoDrop spectrophotometer (Thermo Scientific, NC2000). RNA samples with an RNA integrity number (RIN) > 6.5, as determined by the Agilent 2100 Bioanalyzer System (Agilent Technologies, CA, USA), were used for cDNA construction by the TruSeq RNA Sample Preparation Kit (Illumina, San Diego, CA, USA). Paired-end sequencing was conducted on an Illumina NovaSeq 6,000 platform (Illumina, San Diego, CA, USA) by Shanghai Personal Biotechnology Co. Ltd.

#### Transcriptome Mapping and Genes Analysis

After sequencing, the Illumina paired-end raw reads were processed by Cutadapt (Version 2.7) to remove the 3 'end of the adaptor and the reads with an average mass fraction lower than Q20. The clean reads were matched with the grass carp reference genome (http://www.ncgr.ac.cn/grasscarp/) using HISAT (Version 0.9.1). The FPKM (fragments per kilobases per million fragments) was used to standardize the gene expression levels. Next, genes with |log_2_Foldchange| >1 and adjusted *p*-value <0.05 were defined as differential expressed genes (DEGs) and screened using the DESeq (version 1.39.0). The function and pathway enrichment analyses of all DEGs were performed using the Gene Ontology (GO) database (http://www.geneontology.org/) and Kyoto Encyclopedia of Genes and Genomes Pathway (KEGG) database (http://www.genome.jp/kegg/). A hypergeometric test was used to calculate the *p*-value, and the standard of significant enrichment was *p*-value < 0.05. Furthermore, the GSEA software (v4.1.0, Broad institute, Inc, USA) was used to further investigate the annotated gene sets to reveal the potential impact of protein level on some important KEGG potential pathways of muscle. Results with an absolute value of normalized enrichment score (NES) >1.0 and false discovery rate (FDR) adjusted *p*-value < 0.05 were considered as significantly enriched gene sets.

#### Real-Time Quantitative PCR

The purified total RNA was extracted from dorsal white muscle using Trizol™ reagent (Takara, Dalian, China) according to the manufacturer's instructions. Then, the RNA integrity and quantity were assessed by 1% agarose gel electrophoresis and the 260/280 nm absorbance ratio (NanoDrop® ND-1000, Thermo Fisher Scientific, Waltham, MA, USA). The PrimeScript™ RT reagent Kit (Takara, Dalian, China) was used to reversely transcribe total RNA to cDNA. The real-time quantitative PCR (RT-qPCR) was performed using Unique Aptamer™ qPCR SYBR® Green Master Mix (Novogene, Tianjin, China) on a quantitative thermal cycler (Light Cycler 480II, Roche, Switzerland). Specific primer pairs (Appendix Table 2) were designed for each target gene, which includes muscle fiber type-related genes and the selected DEGs (*ampk-*α, *pprc1, ampd3, cox1, gyk*, and *cryab*) through Oligo 7.0 software based on NCBI known sequence information and transcriptome sequencing results. The RT-qPCR program consisted of an initial activation at 95°C for 5 min, 40 cycles of amplification (95°C for 10 s, 60°C for the 20 s, and 72°C for 20 s). Melting curves were systematically monitored (temperature gradient at 0.5°C/s from 60°C to 95°C) to confirm that only one fragment was amplified. All treatments were conducted with three biological replicates for each gene. The β-actin and ef1α mRNA were used as the internal control. The comparative Ct method (2^−ΔΔCt^), as described by Pfaffl (17), was used to calculate the gene expression values.

### Calculations and Statistical Analysis

The data of initial body weight (IBW), final body weight (FBW), and food intake were used to calculate the following parameters:

Specific growth rate (SGR, %/d) = 100 × [ln (FBW) – ln (IBW)]/[experimental period (d)];

Feed efficiency (FE, %) = 100 × [FBW (g) – IBW (g)]/dry feed intake (g).
Feeding rate (FR, %/d) = 100 × dry feed intake/[experimental days × (FBW + IBW)/2].

Apparent digestibility coefficient of dry matter (ADC_d_, %) =100 × [1– (dietary yttrium content/fecal yttrium content)].

Apparent digestibility coefficient of protein (ADC_p_, %) =100 × [1– (dietary yttrium content/fecal yttrium content) × (fecal protein content/dietary protein content)].

Data were expressed as mean ± SE (standard error, *n* = 3). Statistical differences between treatments were first determined by one-way ANOVA and then by Duncan's multiple test. Data resulting from the dose-response trial were also subjected to orthogonal polynomial contrasts and further subjected to regression analysis to fit the best model if statistical significance was detected (linear, quadratic, or cubic). When a quadratic or cubic regression was noted, the broken-line model was examined as well. The R^2^ was used for the selection of a best-fitted regression model. The correlation between flesh quality and myosin heavy chain isoform expression was analyzed using Pearson's correlation coefficients. All statistical analyses were performed using the software SPSS 22.0 (IBM, Armonk, NY, USA). The difference was considered significant at *p* < 0.05.

## Results

### Growth Performance, Feed Utilization, and Apparent Digestibility Coefficient

The effects of graded levels of dietary protein on growth performance are presented in Table 1. The FBW showed a significant upward trend from SM1 to SM5 and then reached a plateau, which was the highest in the SM5 group (*p* < 0.05). The FE was significantly enhanced by the increasing dietary protein level in a linear model (*p* < 0.05; R^2^ = 0.924). In addition, FR, ADC_p_, and ADC_d_ increased first and then decreased responding to the increasing dietary protein level in quadratic models (R^2^ = 0.738, 0.900, and 0.740), all of which reached the highest values at SM5 group. Based on the broken-line analysis of SGR, the dietary protein requirement for grass carp was estimated to be 38.63% (Appendix Figure 1).

**Table 1:** Effect of dietary protein level on growth performance and feed utilization for grass carp^1^.

**Diets ^**2**^**	**IBW** **(g fish^**−1**^)**	**FBW** **(g fish^**−1**^)**	**SGR** **(% day^**−1**^)**	**FE** **(%)**	**FR** **(%)**	**ADC_**p**_** **(%)**	**ADC_**d**_** **(%)**
SM1	6.79 ± 0.01	11.94 ± 0.31^a^	1.01 ± 0.05^a^	27.52 ± 1.33^a^	2.39 ± 0.01^a^	71.26 ± 1.51^a^	63.53 ± 0.21^a^
SM2	6.78 ± 0.01	13.46 ± 0.77^ab^	1.22 ± 0.10^ab^	42.50 ± 1.44^b^	2.64 ± 0.07^b^	75.51 ± 0.61^b^	66.21 ± 0.81^b^
SM3	6.79 ± 0.01	14.52 ± 0.98^b^	1.35 ± 0.12^b^	45.08 ± 3.55^b^	3.07 ± 0.06^d^	79.01 ± 0.22^c^	67.76 ± 0.83^b^
SM4	6.76 ± 0.01	18.06 ± 0.99^c^	1.75 ± 0.10^c^	57.20 ± 2.55^c^	2.89 ± 0.02^c^	82.49 ± 0.56^d^	68.13 ± 0.83^bc^
SM5	6.78 ± 0.01	23.22 ± 0.05^d^	2.22 ± 0.02^d^	62.16 ± 1.39^cd^	3.24 ± 0.05^e^	88.98 ± 0.35^f^	73.23 ± 0.27^d^
SM6	6.77 ± 0.03	21.95 ± 0.45^d^	2.09 ± 0.04^d^	68.39 ± 1.65^e^	2.90 ± 0.06^c^	85.27 ± 0.26^e^	70.20 ± 0.95^c^
PSE ^3^	0.03	1.19	0.14	3.72	0.08	1.27	1.23
Orthogonal contrast (Pr > F) ^4^							
Linear	0.473	0.000	0.000	0.000	0.000	0.000	0.000
Quadratic	0.391	0.987	0.419	0.056	0.000	0.001	0.033
Cubic	0.301	0.005	0.014	0.494	0.362	0.004	0.127
Regression							
Model ^5^	NOS	2SBL-LL	2SBL-LL	Linear	Quadratic	Quadratic	Quadratic
R^2^		0.659	0.901	0.924	0.738	0.900	0.740
Pr > F ^4^		0.050	0.043	0.000	0.000	0.000	0.000
OPTI ^6^			38.63				

a−f *Different superscript letters within the same row indicate a significant difference (p < 0.05). Data without superscripts indicate no significant differences (p > 0.05)*.

1 *Values are the means of three replicates (n = 3)*.

2 *IBW, initial body weight; FBW, final body weight; SGR, specific growth rate; FE, feed efficiency; FR, feeding rate; ADC_d_, apparent digestibility coefficient of dry matter; ADC_p_, apparent digestibility coefficient of a protein*.

3 *PSE, pooled standard error of treatment means (n = 3)*.

4 *If statistical significance (p < 0.05) was detected, the model that fits best the data was selected*.

5 *NOS, no structure; 2SBL-LL, two slope broken line-linear ascending and linear descending*.

6 *OPTI, optimal protein requirements for grass carp*.

### Whole Body and Dorsal Muscle Composition

As shown in Table 2, the moisture contents in the whole body and dorsal white muscle increased linearly with dietary protein level (*p* < 0.05), whereas the crude lipid and ash contents in the whole body and dorsal white muscle decreased linearly in response to the increasing protein level (*p* < 0.05). Muscle protein content also increased linearly with dietary protein level (R^2^ = 0.333, *p* = 0.012). However, there was no significant difference in the protein content of the whole body (*p* > 0.05).

**Table 2:** Proximate analysis (%; on fresh-weight basis) of whole body and dorsal white muscle of grass carp fed different levels of dietary protein^1^.

**Diets**	**Whole body (%)**				**Dorsal muscle (%)**			
	**Moisture**	**Ash**	**Crude** **protein**	**Crude** **lipid**	**Moisture**	**Ash**	**Crude protein**	**Crude** **lipid**
SM1	70.57 ± 0.13^a^	3.19 ± 0.11^d^	14.72 ± 0.37	10.5 ± 0.47^d^	76.35 ± 0.66^a^	2.36 ± 0.07^d^	18.24 ± 0.24	4.35 ± 0.04^f^
SM2	72.02 ± 0.21^b^	3.02 ± 0.07^cd^	14.60 ± 0.61	9.42 ± 0.22^c^	76.63 ± 0.53^ab^	2.29 ± 0.07^cd^	18.45 ± 0.14	4.13 ± 0.04^e^
SM3	73.16 ± 0.25^c^	2.93 ± 0.01^bc^	14.11 ± 0.06	8.63 ± 0.33^bc^	77.78 ± 0.27^bc^	2.14 ± 0.07^ab^	18.39 ± 0.21	3.94 ± 0.03^d^
SM4	73.56 ± 0.45^c^	2.91 ± 0.07^bc^	14.10 ± 0.28	8.46 ± 0.08^bc^	78.19 ± 0.56^c^	2.08 ± 0.04^b^	18.68 ± 0.11	3.51 ± 0.07^c^
SM5	74.89 ± 0.23^d^	2.69 ± 0.03^b^	14.07 ± 0.26	7.77 ± 0.09^b^	78.30 ± 0.14^c^	1.82 ± 0.08^a^	18.71 ± 0.15	2.06 ± 0.12^b^
SM6	75.94 ± 0.46^e^	2.37 ± 0.12^a^	14.65 ± 0.71	6.64 ± 0.40^a^	78.53 ± 0.16^c^	1.83 ± 0.01^a^	18.72 ± 0.10	1.22 ± 0.01^a^
PSE ^2^	0.45	0.14	0.76	0.53	0.76	0.11	0.28	0.11
Orthogonal contrast (Pr > F) ^3^								
Linear	0.000	0.000	0.635	0.000	0.001	0.000	0.026	0.000
Quadratic	0.432	0.135	0.209	0.838	0.272	0.987	0.613	0.000
Cubic	0.103	0.101	0.605	0.080	0.781	0.416	0.794	0.858
Regression								
Model ^4^	Linear	Linear	NOS	Linear	Linear	Linear	Linear	Linear
R^2^	0.934	0.770		0.847	0.569	0.792	0.333	0.888
Pr > F ^3^	0.000	0.000		0.000	0.000	0.000	0.012	0.000

a−f *Different superscript letters within the same row indicate a significant difference between treatments (p < 0.05). Data without superscripts indicate no significant differences (p >0.05)*.

1 *Values are the means of three replicates (n = 3)*.

2 *PSE, pooled standard error of treatment means (n = 3)*.

3 *If statistical significance (p < 0.05) was detected, the model that fits best the data was selected*.

4 *NOS, no structure*.

### Texture Profile Analysis

The flesh quality parameters of grass carp fed diets containing graded levels of protein are presented in Table 3. The hardness, chewiness, springiness, and cohesiveness in the cooked meat increased first and then decreased responding to the increasing dietary protein level in a quadratic model (R^2^ = 0.803, 0.362, 0.405, and 0.759, respectively), which were the highest in the SM4 group (*p* < 0.05). However, there was no significant difference in resilience among groups (*p* > 0.05). The cooking loss of muscle decreased first and then increased in response to the increasing protein level in a quadratic model (*p* < 0.05, R^2^ = 0.687), which was significantly lower in the SM4 group than SM1, SM5, and SM6 groups (*p* < 0.05) and was not different among SM2, SM3, and SM5. Strikingly, the pH of muscle also increased first and then decreased with protein level in a quadratic model (R^2^ = 0.843) and peaked at the SM4 group.

**Table 3:** Textural profile analysis of dorsal white muscle following dietary protein level treatment ^1^.

**Diets**	**Hardness (g)**	**Chewiness (g)**	**Springiness (cm)**	**Cohesiveness**	**Resilience (g/s)**	**Cooking loss**	**pH**
SM1	675.38 ± 15.04^a^	123.48 ± 7.71^b^	0.41 ± 0.00^a^	0.35 ± 0.01^b^	0.14 ± 0.01	20.20 ± 0.33^b^	5.21 ± 0.01^a^
SM2	722.35 ± 4.23^b^	127.32 ± 2.01^b^	0.41 ± 0.01^a^	0.37 ± 0.01^b^	0.13 ± 0.01	16.19 ± 0.20^a^	5.27 ± 0.01^b^
SM3	791.86 ± 6.88^c^	131.48 ± 2.21^b^	0.43 ± 0.01^a^	0.37 ± 0.01^b^	0.14 ± 0.01	15.54 ± 0.19^a^	5.32 ± 0.01^c^
SM4	877.81 ± 11.76^d^	170.44 ± 1.71^c^	0.48 ± 0.00^b^	0.40 ± 0.01^c^	0.13 ± 0.01	14.84 ± 0.59^a^	5.36 ± 0.01^d^
SM5	812.26 ± 7.19^c^	99.99 ± 1.00^a^	0.44 ± 0.01^a^	0.36 ± 0.01^b^	0.13 ± 0.01	19.28 ± 1.02^b^	5.26 ± 0.02^b^
SM6	692.64 ± 6.61^a^	107.30 ± 2.54^a^	0.41 ± 0.01^a^	0.28 ± 0.01^a^	0.12 ± 0.01	19.54 ± 0.47^b^	5.19 ± 0.01^a^
PSE ^2^	16.21	6.27	0.00	0.00	0.00	0.94	0.00
Orthogonal contrast (Pr > F) ^3^							
Linear	0.000	0.001	0.073	0.000	0.158	0.215	0.236
Quadratic	0.000	0.000	0.001	0.000	0.565	0.000	0.000
Cubic	0.000	0.251	0.006	0.007	0.795	0.012	0.317
Regression							
Model ^4^	Quadratic	Quadratic	Quadratic	Quadratic	NOS	Quadratic	Quadratic
R^2^	0.803	0.362	0.405	0.759		0.687	0.843
Pr > F ^3^	0.000	0.034	0.020	0.000		0.000	0.000

a−d *Different superscripts within the same column indicate a significant difference (p < 0.05). Data without superscripts indicate no significant differences (p > 0.05)*.

1 *Values are means of three replicates (n = 3)*.

2 *PSE, pooled standard error of treatment means (n = 3)*.

3 *If statistical significance (p < 0.05) was detected, the model that fits best the data was selected*.

4 *NOS, no structure*.

### Antioxidant Capacity of Fish Fed With Different Protein Level Diets

**Figure 1:**
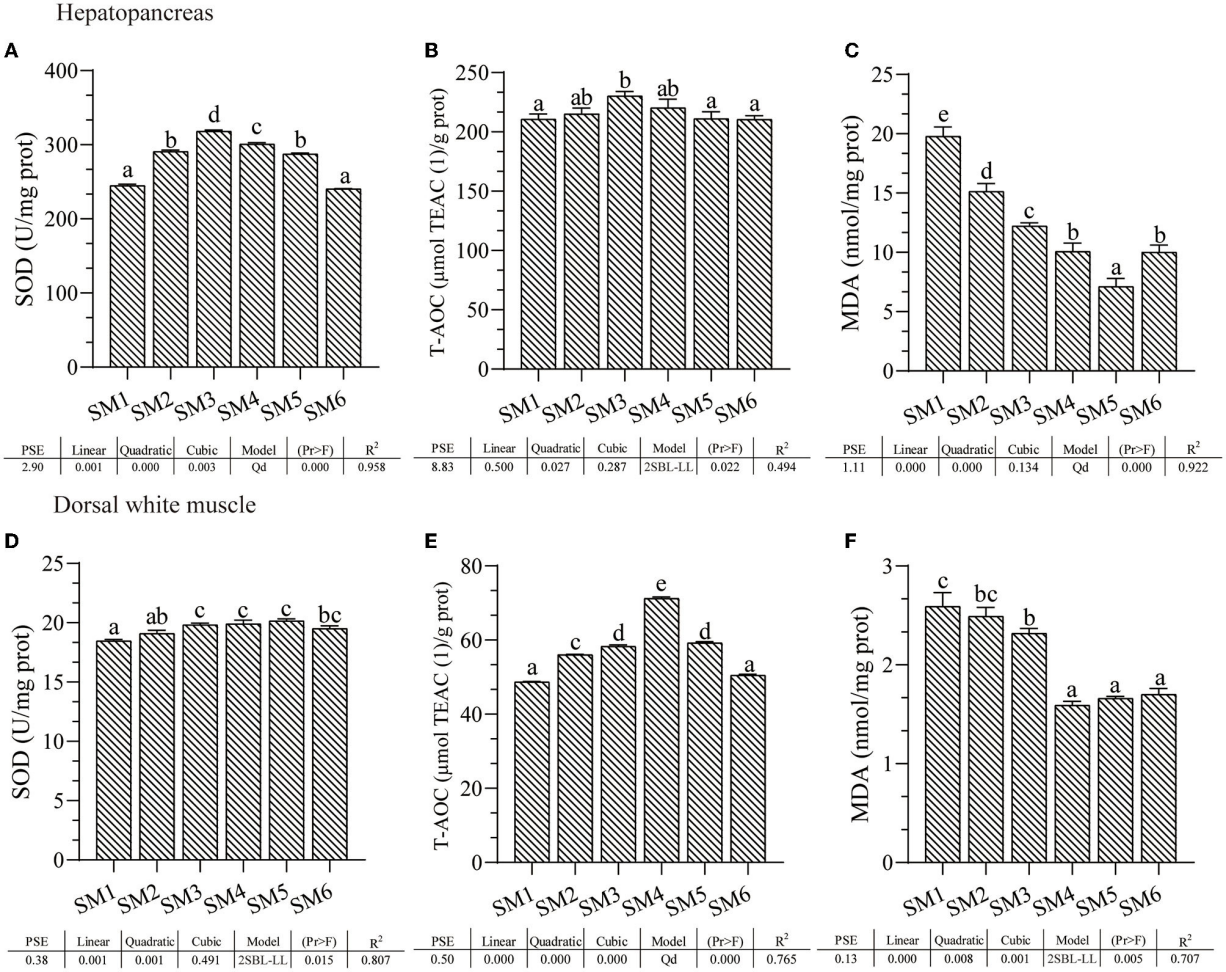
The antioxidant status of grass carp from different dietary protein level groups at the end of the feeding trial. **(A–C)** Superoxide dismutase (SOD) activity, total antioxidant capacity (T-AOC), and malondialdehyde (MDA) content of the hepatopancreas. **(D–F)** SOD, T-AOC, and MDA of dorsal muscle. ^a−*e*^ Different superscript letters within the same row indicate a significant difference (*p* < 0.05). Data without superscripts indicate no significant differences (*p* >0.05). Linear: Linear trend analyzed by orthogonal polynomial contrasts; Quadratic: quadratic trend analyzed by orthogonal polynomial contrasts; Cubic: cubic trend analyzed by orthogonal polynomial contrasts. Model: Qd, quadratic; 2SBL-LL, two slope broken line-linear ascending and linear descending. Pr > F, Probability associated with the F statistic test. TEAC, Trolox equivalent antioxidant capacity.

Activities of SOD, T-AOC, and also MDA contents in hepatopancreas and dorsal white muscle from each dietary treatment are presented in Figure 1. In hepatopancreas, SOD and T-AOC activities significantly increased first and then decreased responding to the increasing dietary protein level in a quadratic model and broken-line model, respectively (R^2^ = 0.958 and 0.494), both of which peaked in the SM3 group (*p* < 0.05); in contrast, the MDA content decreased linearly as protein level increased to SM5 and then increased in SM6 group in a quadratic model (R^2^ = 0.922). Similarly, muscle SOD and T-AOC increased first and then decreased in a broken-line model and quadratic model, respectively (R^2^ = 0.807 and 0.765, respectively) responding to the dietary protein level; T-AOC was the highest in the SM4 protein group, whereas the highest SOD was in the SM5 protein group (*p* < 0.05); similarly, MDA content decreased as dietary protein levels increased in the broken-line model (*p* < 0.05), and MDA content in SM4, SM5, and SM6 groups was the lowest compared with the other three groups (*p* < 0.05).

### Metabolic Enzyme Activities and Microstructure of Dorsal White Muscle Tissue

**Figure 2:**
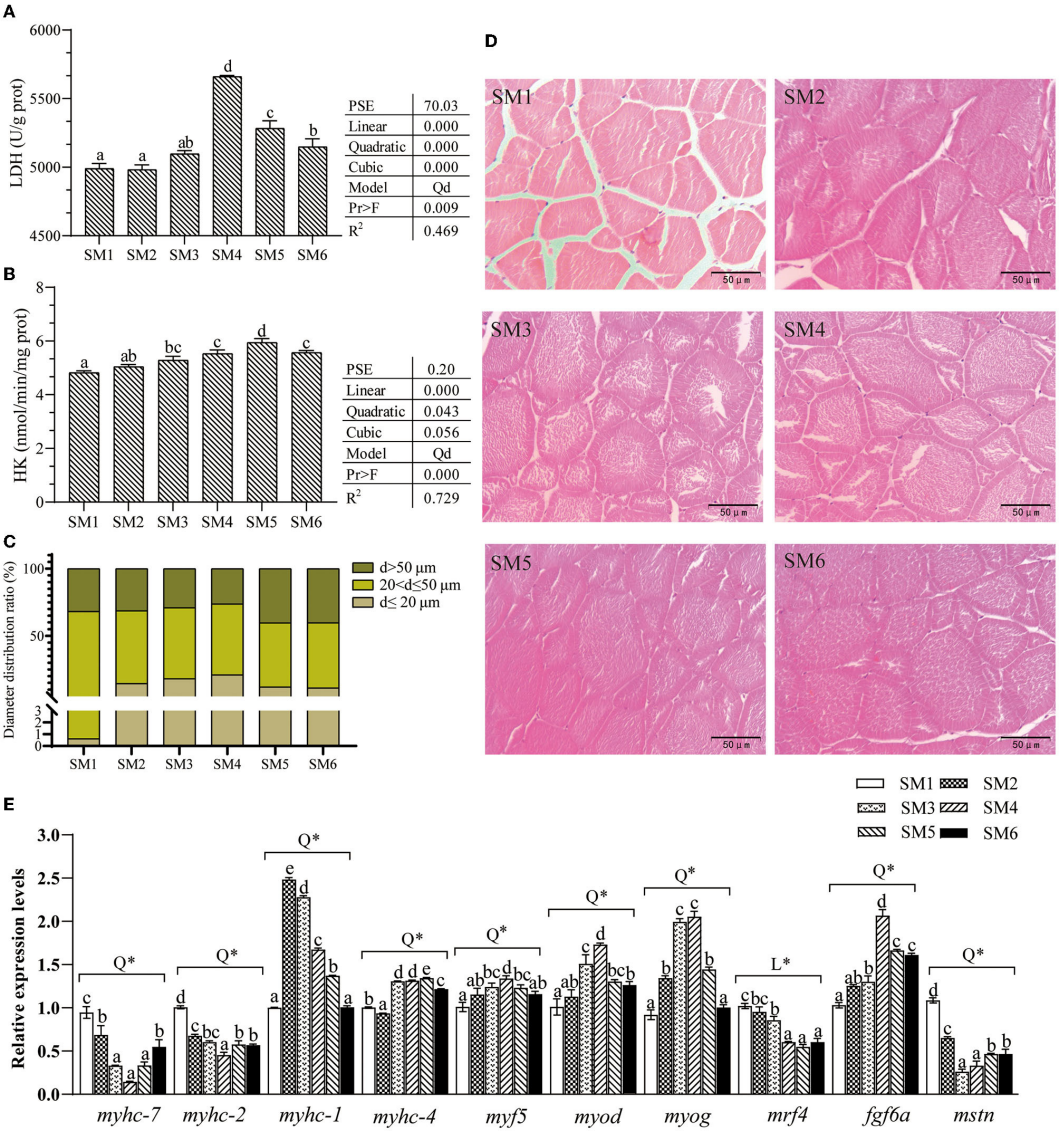
Dietary protein level affected dorsal white muscle structure, metabolic enzyme activities, and gene expression of muscle heavy chain isotypes and myogenic regulatory factors of grass carp. **(A,B)** Activities of LDH and HK (hexokinase), values are the means ± SE (*n* = 3), and different letters above bars indicate the significant difference among treatments (*p* < 0.05). PSE, Pooled standard error of treatment means. Linear: Linear trend analyzed by orthogonal polynomial contrasts; Quadratic: quadratic trend analyzed by orthogonal polynomial contrasts; Cubic: cubic trend analyzed by orthogonal polynomial contrasts. Model: Qd, quadratic. Pr > F, Probability associated with the F statistic test. **(C)** Distribution of the fiber diameter. **(D)** Transverse section microstructure of muscle from the 6 groups. **(E)** Gene expression of myogenic regulatory factors (*myod, myf5, myog*, and *mrf4*), *mstn, fgf6a*, and myosin heavy chain (*myhc-7, myhc-2, myhc-4*, and *myhc-1*). Q (quadratic) and L (linear) means the model of the dependent variable across the graded level of protein by the orthogonal polynomial contrast, * means *p* < 0.01. The cDNAs used to detect genes *myhc-2* and *myhc-4* were diluted 180 times, and *myhc-7* and *myhc-1* were diluted 6 times. Different letters above bars indicate a significant difference (*p* < 0.05).

As shown in Figures 2A,B, the activities of LDH and HK in the white muscle increased first and then decreased in quadratic regression models (R^2^ = 0.469 and 0.729, respectively) as dietary protein level increased. The highest LDH activity in the muscle was observed in the SM4 group, whereas the highest HK activity was observed in the SM5 group (*p* < 0.05).

As shown in Figures 2C,D, dietary protein level affected white muscle microstructure, which shows the different size distribution of muscle fiber diameter. Moreover, the SM4 group had the largest number of small diameter muscle fibers (d < 20 um).

### Effects of Dietary Protein Level on the Expression of Muscle-Related Genes

The gene expression of *myod, myog, myf5, mstn, fgf6a, myhc-7, myhc-2, myhc-4*, and *myhc-1* in the muscle all varied in quadratic models (R^2^ = 0.617, 0.880, 0.654, 0.850, 0.690, 0.842, 0.903, 0.674, and 0.663, respectively) as dietary protein level increased, as presented in Figure 2E. The expression levels of *myhc-7* and *myhc-2* significantly decreased with increasing dietary protein levels up to the SM4 group and increased thereafter (*p* < 0.05). The *myod* and *fgf6a* had the highest expression in the SM4 group. However, *myhc-4* and *myhc-1* mRNA levels were the highest in SM5 and SM2, respectively. Fish fed with the SM4 diet had significantly higher mRNA levels of *myf5, myod*, and *myog* and *fgf6a* in muscle than those fed with other diets (*p* < 0.05). Moreover, the level of *mrf4* mRNA was significantly lower in SM4, SM5, and SM6 groups than SM1 and SM2 groups (*p* < 0.05), which shows a linearly decreasing trend (*p* < 0.001).

### The Correlation Between Meat Quality Traits and *myhc* Types

The correlation coefficients of *myhc* isoforms and flesh quality traits in the grass carp dorsal white muscle are presented in Appendix Table 3. The expression level of *myhc-7* (r = −0.845, −0.671, and −0.693, respectively) and *myhc-2* (r = −0.698, −0.557, and −0.519, respectively) was negatively correlated with hardness, springiness, and pH but positively correlated with cooking loss (r = 0.506 and 0.531). *Myhc-4* had a positive correlation with hardness (r = 0.720) and springiness (r = 0.583) but no significant correlation with cooking loss and pH (*P* > 0.05). Conversely, *myhc-1* showed a positive and negative correlation with pH (*r* = 0.607) and cooking loss (*r* = −0.753), respectively. In addition, chewiness and cohesiveness did not significantly correlate with the expression level of the four *myhc* isoforms (*p* > 0.05). The activities of LDH (r = −0.736 and −0.663, respectively) and HK (r = −0.641 and −0.700, respectively) were negatively correlated with the expression level of *myhc-7* and *myhc-2* while positively correlated with *myhc-4* (r = 0.661 and 0.747).

### Transcription Assembly and Functional Annotation

In this study, 43.05 Mb and 40.91 Mb raw reads were averagely generated in the dorsal white muscle samples from the two experiment groups (SM1 and SM5, respectively), whereas a total of 39.61 Mb and 37.57 Mb of clean bases were obtained from the SM1 and SM5, respectively. The Q30% (the rate of bases in which quality is ≥ 30) is higher than 93%. The alignment rate of clean reads mapped to the reference genome for SM1 and SM5 is 95.13% and 95.13%, respectively (Appendix Table 4). Using NCBI RefSeq as a reference database, a total of 22,447 genes were identified. Among them, 20,282, 7,255, and 14,345 genes were separately functional annotated by Swiss-Prot, GO, and KEGG Orthology databases. Pearson's correlation analysis on the distance between genes using the normalized fragment counts showed that samples are closely correlated with a minimum coefficient of 0.9955 within groups and 0.9721 between samples from different groups, which indicates the high fidelity of sequencing data (Appendix Figure 2A). The first and second components (PC-1 and PC-2) represented the contribution of sample differences. As shown in Appendix Figure 2B, 99.1% of the differences among the six muscle samples came from PC-1.

### Transcriptomic Analysis of the Dorsal White Muscle in Fish From the Low-Protein Diet and High-Protein Diet Groups

#### Gene Expression and Identification of DEGs

**Figure 3:**
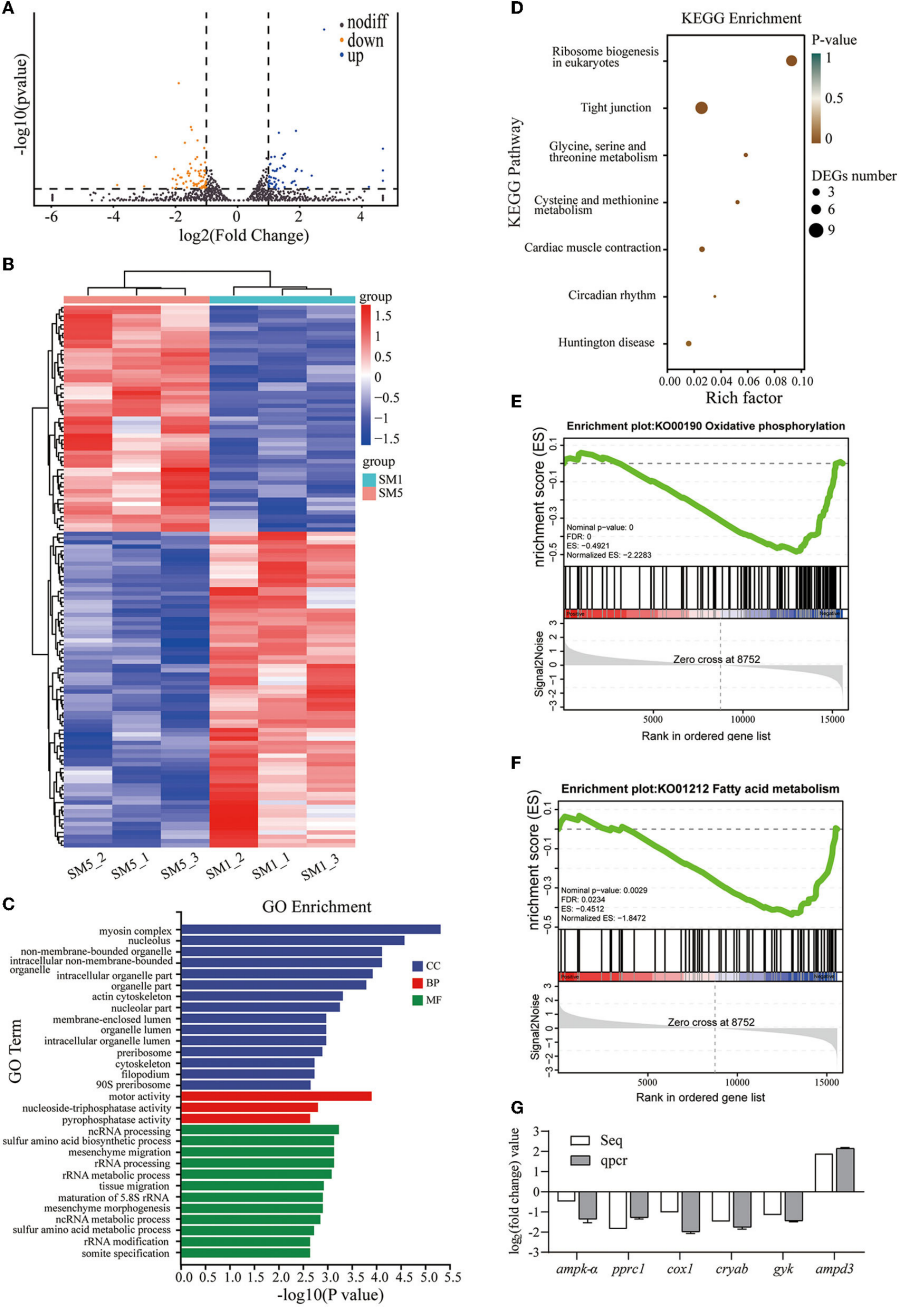
Transcriptomic analysis results of the muscle samples from SM1 and SM5 groups. **(A)** Volcano plot of the differential expressed genes (DEGs). **(B)** Heat map of DEGs. **(C)** Top 30 enriched GO terms of DEGs (*p* < 0.05). **(D)** Differential expressed genes were significantly enriched in KEGG pathways (*p* < 0.05). **(E,F)** Representative two significantly enriched gene sets from gene set enrichment analysis (GSEA) comparing SM5 with SM1, normalized enrichment score (NES) > 1 and FDR < 0.05 were considered statistically significant. **(G)** Comparison between RNA-seq result and qRT-PCR validation result. Seq: high-throughput sequencing. *ampk-*α, AMP-activated protein kinase alpha; *pprc1*, PGC-1-related coactivator; *ampd3*, AMP deaminase 3; *cox*_1_: cytochrome c oxidase subunit 1; *gyk*, Glycerol kinase; *cryab*, Alpha-crystallin B chain.

Comparing the SM5 group with the SM1 group, 127 DEGs, which include 53 upregulated genes and 74 downregulated genes, were identified using DESeq. The distribution of DEGs in the volcano plot (Figure 3A) showed the significant alterations in grass carp dorsal white muscle transcriptome induced by the low- or high-protein level diets. In addition, hierarchical clustering of significantly up- and downregulated genes was subsequently conducted and is depicted as an expression heat map (Figure 3B).

#### GO, KEGG, and GSEA Enrichment Analysis of DEGs

The gene ontology (GO) enrichment analysis showed that DEGs were involved in biological process (BP), cellular component (CC), and molecular function (MF), and the top 30 enriched GO terms are shown in Figure 3C (*p*-value < 0.05). In terms of BP, the main DEGs were involved in amino acid metabolism and ribosome biogenesis processes (including sulfur amino acid metabolic process (GO: 0000096), rRNA processing (GO: 0006364), rRNA modification (GO: 0000154), and so on). Moreover, in a CC, GO terms related to muscle cell structure were enriched, which include myosin complex (GO: 0016459), cytoskeleton (GO: 0005856), actin cytoskeleton (GO: 0015629), organelle part (GO: 0044422), and nucleolus (GO: 0005730). Other enriched GO terms were related to MF, which include motor activity (GO: 0003774), pyrophosphatase activity (GO: 0016462), and nucleoside triphosphatase activity (GO: 0017111).

As shown in Figure 3D, the KEGG pathway analysis (*p*-value < 0.05) revealed the enrichment of pathways related to ribosome biogenesis in eukaryotes (ko03008), glycine, serine, and threonine metabolism (ko00260), and cysteine and methionine metabolism (ko00270). Notably, myosin heavy chain, fast skeletal muscle, and heat shock 70 kDa protein 4 (HSPA4) were significantly enriched (*p* < 0.05) in the tight junction pathways (the DEGs involved in the KEGG pathway are listed in Appendix Table 5), which play important roles in the cytoskeleton and myofibril structure. In addition, pathways that include cardiac muscle contraction (ko04260), circadian rhythm (ko04710), and Huntington's disease (ko05016) were also significantly enriched (*p* < 0.05).

Gene set enrichment analysis showed that oxidative phosphorylation was significantly downregulated in dorsal muscle tissues of SM5 with a normalized enrichment score (NES) of −2.2283 and FDR of 0.000 (Figure 3E). Moreover, fatty acid metabolism gene sets were downregulated in the SM5 group, with a normalized enrichment score (NES) of −1.8472 and FDR of 0.0234 (Figure 3F). Heat maps of representative enriched genes of each gene set are listed in Appendix Figure 3.

#### Validation of Transcriptome Data by QPCR

We selected six genes (*ampk-*α, *pprc1, ampd3, cox11, gyk*, and *cryab*) for qPCR analysis to validate the RNA-seq results obtained. As shown in Figure 3G, the qPCR expression patterns of the selected genes were consistent with the RNA-seq analysis results.

## Discussion

### Optimal Dietary Protein Levels Improved Fish Growth and Its Requirement in Juvenile Grass Carp

This study revealed that the growth performance and feed utilization of juvenile grass carp were influenced by dietary protein level, which confirmed the previous results (2). Based on the specific growth rate, the optimal dietary protein level for grass carp juvenile (initial body weight at 6.8 g) was estimated to be 38.63% using broken-line analysis, which was slightly lower than the requirement of 410–430 g kg^−1^ diet for fry (0.15–0.2 g) reported by Dabrowski (2) and 400 g kg^−1^ for grass carp fry (4.27 ± 0.01 g) reported by Jin et al. (3). This variation in dietary protein need might be related to the different life-history stages, as direct proof showed a heavy weight of the fish require less protein (18). Also, it was suggested that the excess dietary protein intake does not further improve the weight gain of fish (3), which is confirmed in this study. This may be explained by the decreased ADC of protein in the SM6 group in this study. In addition, the excess of amino acids absorbed would cost additional energy during metabolism (18). This indicates that soybean meal could be used as an excellent protein source for the grass carp diet.

The carcass composition was markedly related to the dietary protein level, especially the lipid content in the whole body, and muscle decreased linearly as dietary protein level increased, which was supported by another study on grass carp by Jin et al. (3). This indicates that dietary protein plays an important role not only in growth but also in meat quality regulation (4). Thus, we next assayed the effects of protein level on the flesh quality of grass carp.

### Diet Protein Level Affected the Flesh Quality of Grass Carp

The meat quality of cultured fish has got more attention due to the improvement in people's living levels. It is extensively considered that hardness, an important indicator in meat quality evaluation, is usually positively correlated with flesh quality in farmed fish (13). The flesh quality parameters such as hardness, cooking loss, and pH varied in response to the dietary protein level, which was in accordance with the previous results to some extent (4, 19). The changes in physical indicators of meat may be related to the alterations in the metabolic and morphological properties of muscle fibers (19, 20).

Intramuscular fat (IMF) content has been considered to be correlated with the tenderness of the meat, but the specific correlation is still equivocal (20). In this study, the hardness of muscle was the highest in SM4, and decreasing levels of IMF led to a first increase and then reduction in hardness, which suggests that meat tenderness is not only determined by IMF, but also by other factors. It is reported that in addition to IMF, unique characteristics of muscle fibers, such as fiber diameter and metabolic mode, jointly determine the hardness of meat (20), which agrees with the results in this study. Moreover, the expression of *myhc* isoforms was strongly associated with meat hardness in livestock (7), which seems to be equally relevant in fish. Recently, transcriptomics analysis showed that changes in the hardness of the dorsal muscles of grass carp were related to alterations of *myhc* isoform expression level (13). Our study confirmed this. Correlation analysis between *myhc* isoform and hardness showed that *myhc-7* (*r* = −0.845) and *myhc-2* (*r* = −0.698) were negatively correlated with hardness, whereas *myhc*-*4* had a positive correlation with hardness (*r* = 0.720) in this study, which are consistent with previous results that pork with a higher proportion of type IIB fibers exhibits greater hardness (7). Furthermore, when analyzing the fiber diameter, significant differences were observed between the groups in this study. The fiber diameter is negatively correlated with fish muscle hardness (15), which may explain the higher meat hardness value observed in the SM4 group in our study as it showed a higher proportion of smaller diameter muscle fibers. Moreover, it was suggested that differences in metabolic characteristics connected with *myhc* isoforms (6), thereby affecting meat quality. It has been shown that muscle fibers with a higher percentage of *myhc-4* exhibit a higher glycolytic capacity (21). This observation is consistent with our results that the highest LDH and HK activities were detected in SM4 and SM5 groups, respectively. In living muscle, lactic acid produced by anaerobic glycolysis is shunted to the liver through blood circulation for degradation (22), which explained that high LDH enzyme activity is accompanied by a high pH value in the SM4 group. Therefore, the alteration in hardness values, shown in this study, is probably due to the changes in IMF content and muscle fiber structure in dorsal white muscle of grass carp. It is worth emphasizing that from the results of this study, dietary protein may control the type of myosin heavy chain and improve meat quality.

### Diet Protein Levels Affected the Antioxidant Capacity of the Tissue

The antioxidant system is a critical barrier for maintaining aquatic animals' health and can be altered by the intake of nutrients (4). Peroxidation in muscle food is a serious problem for the food industry since it leads to myofibrillar protein fragmentation and decrease of solubility, which affects the quality of meat (23). Optimal dietary protein level significantly decreased muscle MDA content while increasing the SOD activity, T-AOC, and the mRNA levels of antioxidant enzyme (24, 25). In contrast, Huang et al. (26) reported that a low-protein diet inhibited the antioxidative enzyme activities in rat muscle. Consistent with this, appropriate dietary protein levels increased SOD activity, T-AOC, and reduced lipid peroxidation in this study. In addition, our results also showed that excessive protein intake would reduce the SOD activity and T-AOC of grass carp. This may suggest that the adverse effects of excessive soybean meal on the antioxidant capacity of fish, which was consistent with the findings in turbot (5). Therefore, we surmise that the improvement in meat quality of grass carp fed with appropriate protein content is partly due to an improved antioxidant capacity in muscle tissue.

### Protein Levels in the Diet-Regulated Myosin Heavy Chain Isoforms (*myhcs*) Gene Through Myogenic Regulatory Factors (MRFs) Gene Expression

Previous studies on rainbow trout showed that a diet rich in soybean meal led to a significantly increased fast *myhc* mRNA level in skeletal muscle (27). Expectantly, we found that optimal protein level promoted *myhc-1* and -*4* expression while downregulating the expression levels of *myhc-7* and *-2*.

Currently, the mechanism of the nutrients inducing *myhc* isoform expression is getting rising attention, during which the regulatory factors are widely investigated. Our previous research has demonstrated that partial replacement of rapeseed meal and cottonseed meal with DDGS downregulated the expression levels of *myod* and *myf5* and also changed the *myhc* expression level in grass carp muscle (10, 15). Consistently, Alami-Durante et al. (27) reported that the alterations in *myod* expression might be due to the changes in dietary plant protein sources and amino acid profiles. In this study, the transcription of *myf5, myod*, and *myog* varied with the dietary protein level in quadratic models (R^2^ = 0.790, 0.617, and 0.880, respectively), whereas *mrf4* varied in a linear model (R^2^ = 0.804), which indicated that the MRFs expression responded to the dietary nutrient intake. Moreover, fish *myhc* shows interchangeability and is controlled by various transcription factors such as MRFs (8). In this study, the fish fed with diets containing high-protein levels had a higher expression of *myf5* and recruited more new fiber, which suggested that a higher dietary protein caused a potential higher contribution of fiber recruitment (hyperplasia), as reported in meager (*Argyrosomus regius*) (12). However, this complex regulatory mechanism of *myf5* on *myhc* specific expression is still unclear in fish. In zebrafish myogenesis program, *myod* can activate fast *myhc* transcription (8). This study partially confirmed this as the grass carp muscle *myhc-1* and *myhc-4* mRNA levels were positively correlated with *myod* expression. Consistently, Ekmark et al. (28) reported that the expression of *myhc-1* and -*4* was restricted by *myod cis*-regulatory regions. These results suggest that dietary protein itself or its metabolites may regulate *myhc* expression pattern, which is related to the expression of *myod*. In addition, *myog* may be an indicator of muscle hypertrophy and participate in the process of myotube fusion and growth (11). Based on the muscle HE staining section, higher levels of protein can trigger muscle fiber hypertrophy with larger fiber diameter, which is also evidenced by the higher expression of the *myog* gene in the muscle. Meanwhile, the *mrf4* linearly decreased as the dietary protein level increased, which may be explained by the *mrf4* acted as a negative regulator of fiber hypertrophy as demonstrated by Moretti et al. (29) using an RNA interference (RNAi) approach. In addition, *fgf6a* and *mstn* possibly play a crucial role in the regulation of muscle proliferation and hypertrophy in fish (30, 31), which agree with the *mstn* and *fgf6a* expression level responding to the muscle growth in this study.

### Differential Responses of the Muscle Transcriptome to Low- and High-Protein Diets in Grass Carp

Interestingly, the *cryab* was used as a valuable biomarker of marbling in livestock (32), and the expression of *cryab* significantly decreased in a fed high-protein diet in this study. In addition, KEGG analysis demonstrated that *myhc* isoform and *hsp74* were significantly enriched in the tight junction in this study, which was once considered to be tightly related to meat hardness (32). Regretfully, due to the homology differences in myosin heavy chain nucleotide sequences in fish and higher vertebrates and the reduced isoform discovery effect resulting from the short read-sequencing in the transcriptome (33), the subtypes (*myh1, myh2*, and *myh4*) of myosin heavy chain, fast skeletal muscle, could not be annotated in grass carp transcripts in this study and previous studies (34). Instead, the expression status of *myhc* isoforms presented in Figure 2E and the relation between meat hardness and *myhc* isoforms in Appendix Table 3 clearly supported that the *myhc* isoform profile affected muscle hardness. Commonly, interconversion between *myhc* isoform within the muscle can be induced by nutritional status. It is well confirmed that both adenosine 5‘-monophosphate (AMP)-activated protein kinase (*ampk*) and peroxisome proliferator-activated receptor gamma coactivator-related protein 1 (*pprc1*) play a crucial role in the conversion between fast and slow *myhc* and in the energy generation and consumption (21, 35). Ulteriorly, the transcription level of *ampk* (the log_2_(fold change) = −0.48144) and *pprc1* was significantly reduced in the high-protein diet (SM5) in the study. Such effects appear to be mediated by the changes in intracellular AMP/ATP ratio, as the gene expression of AMP-deaminase 3 (AMPD3), an enzyme to catalyze AMP to produce IMP and restrict the accumulation of intracellular AMP (36), was significantly upregulated in the transcriptome of fish from the high-protein diet group compared with the low-protein diet group in this study. Moreover, it is reported that *myhc-4* and *myhc-1* have the highest isometric ATP consumption rate (37). In addition, to avoid missing essential genes with minor fold changes, we conducted GSEA to gain a comprehensive understanding of potential mechanisms responses to a high-protein diet. The GSEA results showed that the oxidative phosphorylation pathway was downregulated in a high-protein diet, which was the key pathway of ATP production in the presence of oxygen (38), which further suggests the energy utilization pattern of muscle fibers changed. Particularly, our results showed that several DEGs were involved in regulating the electron transfer chain. Among these genes, the cytochrome c oxidase (*cox1*_1_) was downregulated in the high-protein diet, which transfers electrons from cytochrome *c* (Cyt. *c*) to O_2_ and conduces to generate proton gradients to drive ATP synthesis (39). Above all, grass carp skeletal muscle may adapt to the dietary protein level by a diverse array of signaling transduction cascades, such as changing its own energy metabolism of muscle fibers, which in turn promote the fast myosin heavy chain expression, thus affecting the flesh quality.

The high-protein diet-induced lower lipid content in muscle and whole body may be explained by the transcriptome results in this study. It has been demonstrated that prolonged high-protein diet decreased the lipid accumulation by depression of lipogenic genes expression in the liver in black seabream (*Ancherythroculter nigrocauda*) (40), which seems to be similar in skeletal muscle in this study, as certain DEGs on lipid synthesis were screen out between different dietary protein groups. In the white muscle of fish subjected to a high-protein diet, both the expression of glycerol kinase (GyK), an important enzyme for producing glycerol 3-phosphate in lipogenesis (41), and the mRNA transcription of nocturnin (NOC), a circadian-regulated protein involved in adipogenesis through cooperation with PPAR-γ (42), were downregulated. Furthermore, GSEA showed that most related genes (CPT-I, CPT-II, SCD, and ACSL, etc.) in the fatty acid metabolism signaling pathway were downregulated in the SM5 group, which altered the rates of fatty acids biosynthesis (40). Thus, considering the gene expression alteration related to fatty acid uptake, storage, and oxidation, it could be speculated that dietary protein levels lead to major differences in the content of IMF and give rise to crucial consequences for meat nutrition and meat quality.

## Conclusion

In brief, the optimal dietary protein level was determined to be 38.63% for the best growth performance of juvenile grass carp when SM was used as the protein source. This study demonstrated that appropriate dietary protein levels effectively enhanced the antioxidant capacity in hepatopancreas and muscles, and also the meat quality of grass carp. Moreover, meat quality differing in the mRNA transcript of *myhc-7, myhc-2, myhc-1*, and *myhc*-*4* responded to the dietary protein levels by changing the metabolic status, antioxidant capacity, and differentially expressing MRFs (*myod, myog mrf4 myf5*). The dorsal white muscle transcriptome analysis further revealed that, compared with the fish fed with the low-protein diet (SM1), those with a high-protein diet (SM5) showed downregulated DEGs in the fatty acid metabolism signaling pathway and oxidative phosphorylation pathway, and some DEGs involved in amino acid metabolism and muscle cell structure.

## Data Availability Statement

The datasets presented in this study can be found in online repositories. The names of the repository/repositories and accession number(s) can be found in the article/Supplementary Material. Transcriptome sequencing data have been deposited at the National Center for Biotechnology Information (NCBI) (BioProject: PRJNA789674).

## Ethics Statement

The animal study was reviewed and approved by Animal Care and Use Ethics Committee of Huazhong Agricultural University.

## Author Contributions

XW: conceptualization, writing-original draft, and investigation. GL: visualization. SX: conceptualization. LP: writing-review and editing. QT: conceptualization, writing-review and editing, supervision, and investigation. All authors contributed to the article and approved the submitted version.

## Funding

This work was financially supported by the National Key R&D Program of China (2019YFD0900200) and the National Natural Science Foundation of China (Grant No. 32072950).

## Conflict of Interest

The authors declare that the research was conducted in the absence of any commercial or financial relationships that could be construed as a potential conflict of interest.

## Publisher's Note

All claims expressed in this article are solely those of the authors and do not necessarily represent those of their affiliated organizations, or those of the publisher, the editors and the reviewers. Any product that may be evaluated in this article, or claim that may be made by its manufacturer, is not guaranteed or endorsed by the publisher.
