# Supplementary material for: Growth and Meat Quality of Grass Carp (Ctenopharyngodon idellus) Responded to Dietary Protein (Soybean Meal) Level Through the Muscle Metabolism and Gene Expression of Myosin Heavy Chains
Source: Front Nutr. 2022 Mar 28;9:833924. doi: 10.3389/fnut.2022.833924 (PMC8996190; doi:10.3389/fnut.2022.833924)
Supplement: Supplementary file 1 [file Data_Sheet_1.pdf]

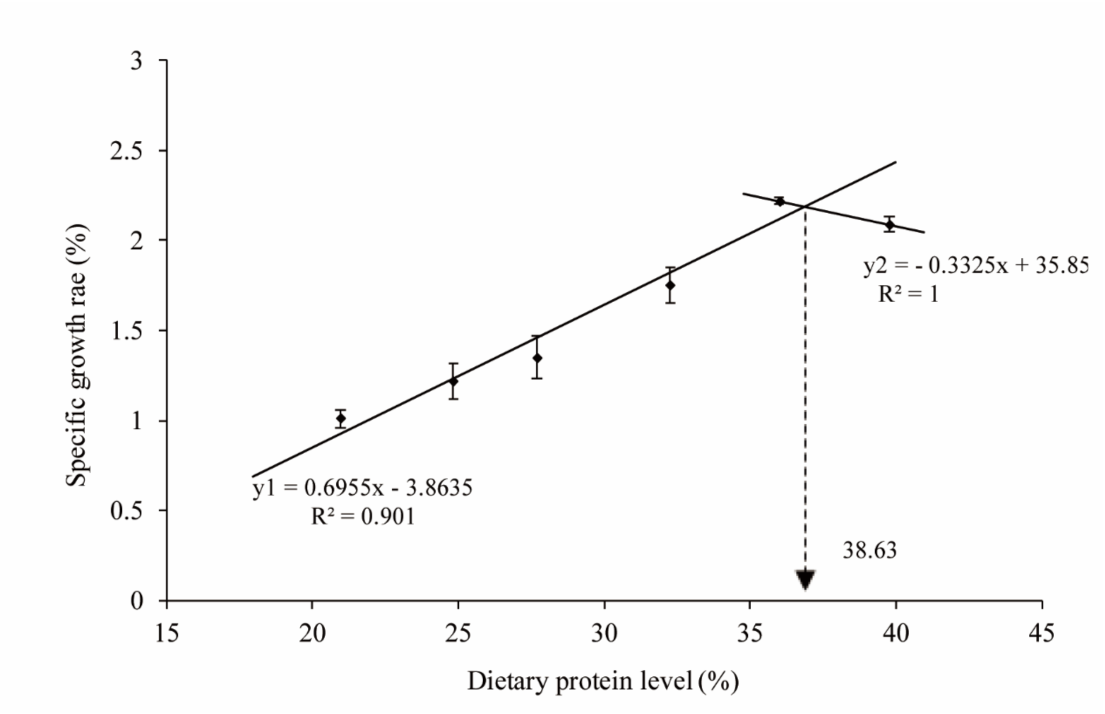

Appendix Fig 1. Broken-line analysis based on the specific growth rate of grass carp in response to various protein levels, respectively. X in the equations represents the dietary protein level (%).

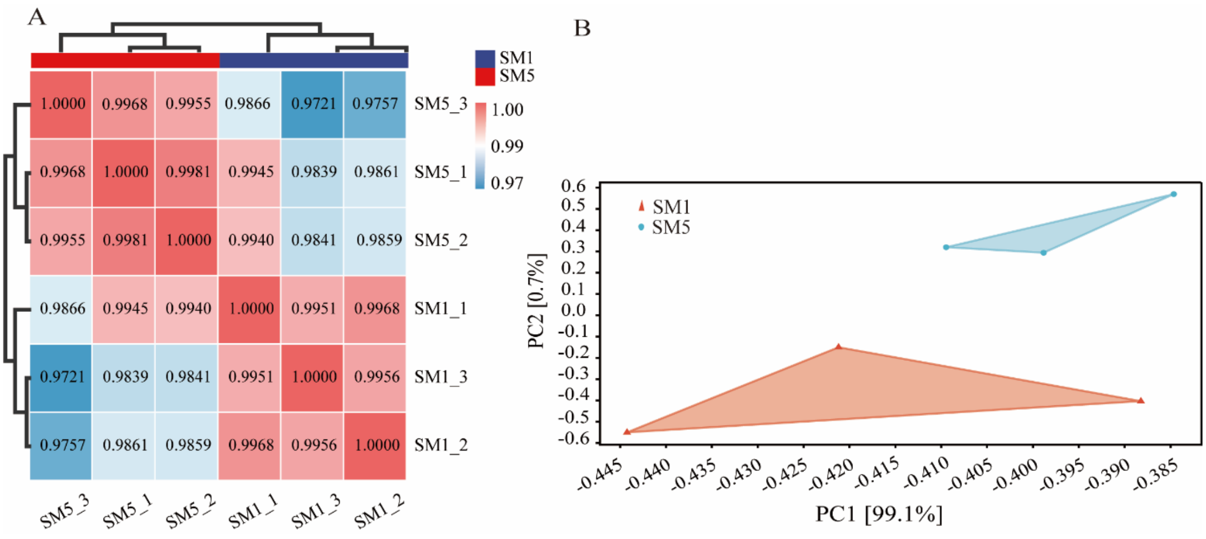

Appendix Fig 2. Heat map and PCA analysis of the tested samples. A. Correlation heat map of muscle samples, and numbers in the boxes represent the Pearson's correlation coefficient between two corresponding samples. B. PCA analysis of the tested samples.

A. Oxidative phosphorylation (KO00190)

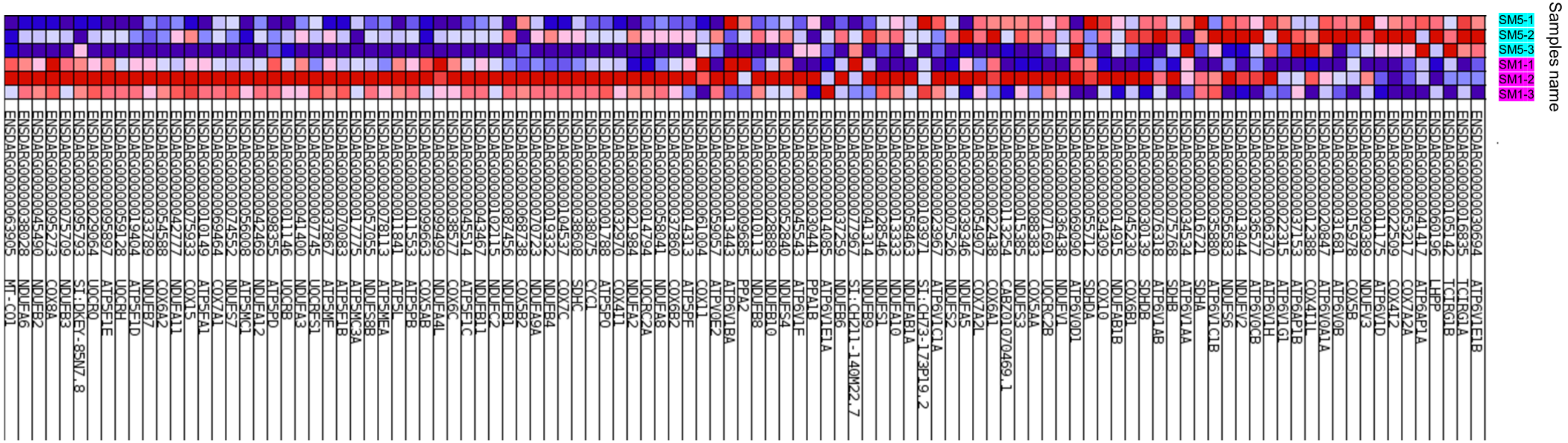

*   The color transition from blue to red indicates that the gene expression is from low to high.

B. Fatty acid metabolism (KO01212)

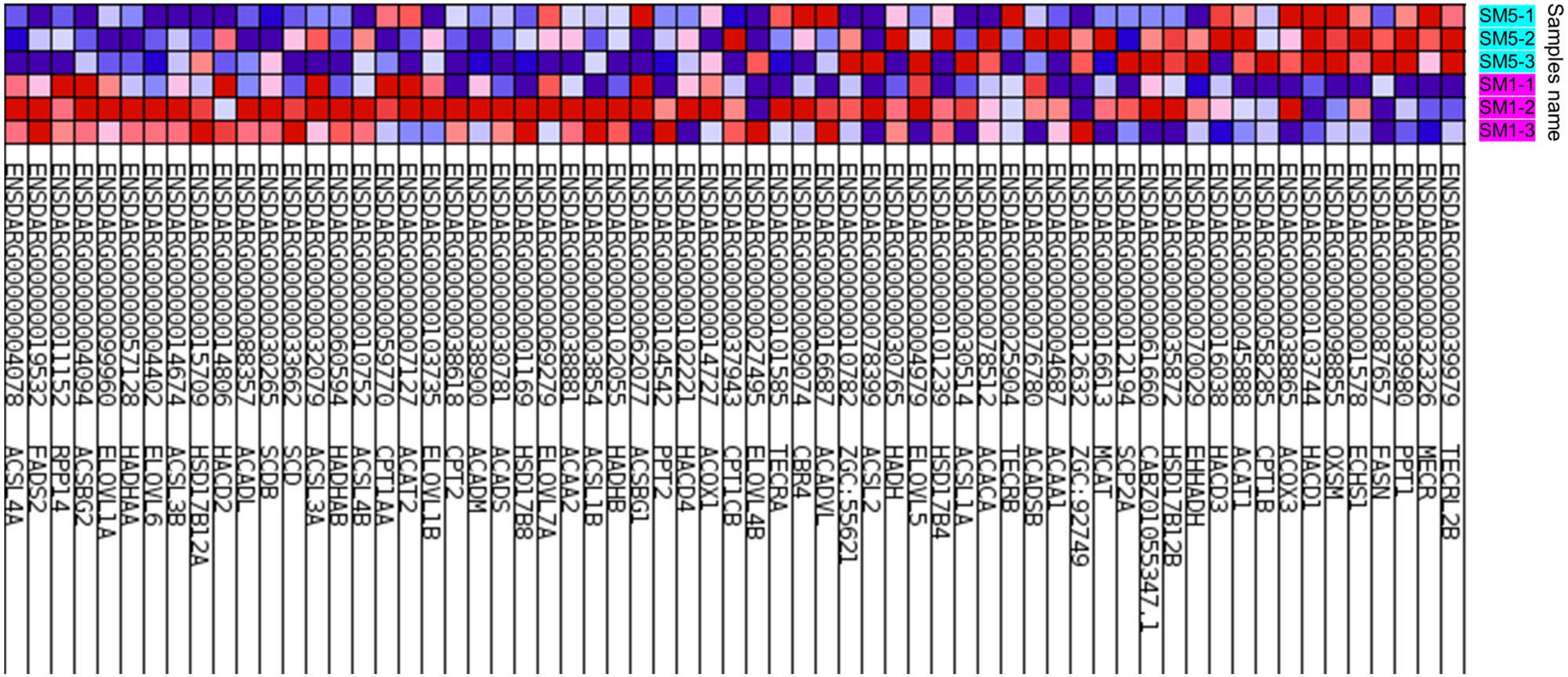

* The color transition from blue to red indicates that the gene expression is from low to high.

Appendix Fig 3. Heat maps of representative enriched genes of each gene set from GSEA.

Appendix Table 1. Formulation and proximate composition of the experimental diets (% in dry matter).

Ingredients	Dietary protein levels (% in dry matter)					
	SM1	SM2	SM3	SM4	SM5	SM6
Soybean meal	40.80	48.60	56.50	64.40	72.20	80.00
Soy oil : Fish oil (1:1)	6.40	6.32	6.25	6.16	6.10	6.00
Corn starch	14.50	11.60	8.70	5.80	2.90	0.00
Microcrystalline cellulose	34.90	30.08	25.15	20.24	15.40	10.60
Vitamin and mineral premix[1]	1.00	1.00	1.00	1.00	1.00	1.00
Calcium biphosphate	1.80	1.80	1.80	1.80	1.80	1.80
Choline chloride (50%)	0.50	0.50	0.50	0.50	0.50	0.50
Yttrium oxide	0.10	0.10	0.10	0.10	0.10	0.10
Proximate composition						
Moisture (%)	5.40	5.42	5.95	5.58	6.07	5.32
Crude protein (%)	20.95	24.83	27.68	32.26	35.98	39.76
Crude lipid (%)	6.96	6.72	6.87	7.21	7.10	7.01
Total ash (%)	4.37	4.91	5.41	5.95	6.45	7.17

[1] Per kilogram of vitamin and mineral premix containing: L-ascorbate-2-monophosphate (35%), 900mg; vitamin E, 450mg; inositol, 225mg; nicotinamide, 120mg; calcium pantothenate, 60mg; vitamin A, 30mg; vitamin K3, 30mg; vitamin B2, 22.5mg; vitamin B6, 22.5mg; vitamin D3, 15mg; vitamin B1, 15mg; folic acid, 15mg; vitamin B12, 120 μg; biotin, 3mg; ferrous sulfate monohydrate, 300mg; zinc sulfate/sulphate monohydrate, 200mg; Sodium chloride, 100mg; manganese sulphate, 25mg; copper (II) sulfate pentahydrate, 30mg; cobaltous chloride (10% Co), 5mg; sodium selenite (10% Se) , 5mg; potassium iodate (2.9%), 3mg; magnesium sulphate, 900mg.

Appendix Table 2. Real-time PCR primer sequences.

Genes [1]	Forward primer (5′-3′)	Reverse primer (5′-3′)	Accession number
myod	ATGGAGTTGTCGGATATTCCCTTC	GCGGTCAGCGTTGGTTGTT	MG544985
myog	TTACGAAGGCGGCGATAACTT	TGGTGAGGAGACATGGACAGA	JQ793897
myf5	GTGCCTGTGCCTCATCTCCT	AATGCGTGGTTCACCTTCTTCA	GU290227
mrf4	TCGCTCCTGTATTGATGTTGATGA	GCTCCTGTCTCGCATTCGTT	KT899334
fgf6a	CGCATACGAGTCTTCCAT	CCTACGAGAACATCCAACA	MK050993
mstn	CTGACGCCAAGTTCCACATACA	CGACTCTGCTTCAAGTTCTTCTCT	KP719016
myhc-7	AACTGCGCTGTAACGGTGTA	AGTGTGCCCAAACCTGTACT	MW113233
myhc-2	ACAGTGGCCAGCATTGATGA	TCCGCAGAGTTCAAACCCAA	MW113235
myhc-4	ACTCCGCTGACATGCTGAAA	TGTCCAGCACACCAATGAAGA	MW113236
myhc-1	TTCCGTTGTTGTGTCAGGCT	TACTGGATGACGCGTTTGGT	MW113234
β-actin	TATGTTGGTGACGAGGCTCA	GCAGCTCGTTGTAGAAGGTG	M25013
ef1α	TGACTGTGCCGTGCTGAT	CGCTGACTTCCTTGGTGATT	GQ266394
Primer sequences of validate the transcriptome candidate genes [2]			
ampk-α	TAGTGGCCGAGACTCAACCG	TCTGGCTACGGATTCCCAGG	
pprc1	AAGAGGAGGAAGATAGGGAT	CATGTGTTTTACTAAATCGCT	
ampd3	ACTAATGGCTGAGAAAGTGT	GAAATTGATTGGGATCGCAT	
cox1	GCATCCGTAGACCTAACAAT	CTGTATTTCATAGGGCGTAG	
gyk	TGGGGTGACAAATCAGAGGG	GCCAGTCTTGTGCTTGAGGT	
cryab	GTTGGAGATTATGTTGTGGT	TTACTAGCATTCCTTCCGGT	

[1] myhc-7: myosin heavy chain 7, myhc-2: myosin heavy chain 2, myhc-1: myosin heavy chain 1, myhc-4: myosin heavy chain 4, ampk-α: AMP-activated protein kinase alpha. pprc1: PGC-1-related coactivator. ampd3: AMP deaminase 3. cox1: cytochrome c oxidase subunit 1. gyk: Glycerol kinase. cryab: Alpha-crystallin B chain.

[2] Design primers based on the sequence determined by the transcriptome.

Appendix Table 3. Correlation coefficients (r) between myosin heavy chain (*myhc*) isoforms and meat quality.

	myhc 7	*myhc 2*	*myhc 1*	*myhc 4*
Hardness	-0.845***	-0.698***	ns	0.720***
Chewiness	ns	ns	ns	ns
Springiness	-0.671**	-0.557*	ns	0.583*
Cohesiveness	ns	ns	ns	ns
Cooking loss	0.506*	0.531*	-0.753***	ns
pH	-0.693***	-0.519*	0.607**	ns
LDH	-0.736**	-0.663**	ns	0.661**
HK	-0.641**	-0.700**	ns	0.747***

Significance of correlations: n.s., not significant, $P > 0.05$; *, $P < 0.05$; **, $P < 0.01$; ***, $P<0.001$.

LDH (lactate dehydrogenase) and HK (hexokinase).

Appendix Table 4. Summarized sequencing information of grass carp muscle transcriptomes.

Samples	SM1 group1	SM1 group 2	SM1 group 3	SM5 group 1	SM5 group 2	SM5 group 3
Raw reads (Mb)	42.78	42.51	43.85	38.35	40.30	44.08
≥Q30 reads (%)	93.61	93.54	93.33	93.44	93.22	93.51
Clean reads (Mb)	39.39	39.00	40.44	35.28	36.89	40.54
Total mapped (%)	95.25	95.03	95.11	95.40	94.83	95.15

Appendix Table 5. The DEGs involved in ribosome biogenesis in eukaryotes, tight junction, glycine, serine and threonine metabolism, cysteine and methionine metabolism, cardiac muscle contraction, circadian rhythm and huntington disease pathway.

Pathway ID	Pathway	P-value	Adjust P-value	DEGs name
ko03008	Ribosome biogenesis in eukaryotes	2.29623E-08	3.00807E-06	H/ACA ribonucleoprotein complex subunit 1;<br>WD repeat-containing protein 75;<br>rRNA 2'-O-methyltransferase fibrillarin;<br>Midasin;<br>Nucleolar protein 6;<br>Periodic tryptophan protein 2 homolog;<br>RNA-binding protein 28;<br>RNA exonuclease 5
ko04530	Tight junction	0.00012929	0.00846852	Myosin heavy chain, fast skeletal muscle;<br>Heat shock 70 kDa protein 4 (HSPA4);
ko00260	Glycine, serine and threonine metabolism	0.002904666	0.1268371	Betaine--homocysteine S-methyltransferase 1;<br>Cystathionine beta-synthase;
ko00270	Cysteine and methionine metabolism	0.003985126	0.130512871	Betaine--homocysteine S-methyltransferase 1;<br>Cystathionine beta-synthase;
ko04260	Cardiac muscle	0.010632478	0.278570915	Actin, alpha cardiac; Actin, alpha skeletal muscle 2;

	contraction			Cytochrome c oxidase subunit 1;
ko0471<br>0	Circadian rhythm	0.039451<br>288	0.85293767<br>8	Nuclear receptor ROR-beta;<br>Nuclear receptor ROR-alpha;
ko0501<br>6	Huntington disease	0.049854<br>62	0.85293767<br>8	Protein-glutamine gamma-glutamyltransferase 2;<br>Dynein heavy chain 6;<br>Cytochrome c oxidase subunit 1;
